# Supplementary figures and images for: Bayesian inference of neuronal assemblies
Source: PLoS Comput Biol. 2019 Oct 31;15(10):e1007481. doi: 10.1371/journal.pcbi.1007481 (PMC6850560; doi:10.1371/journal.pcbi.1007481)

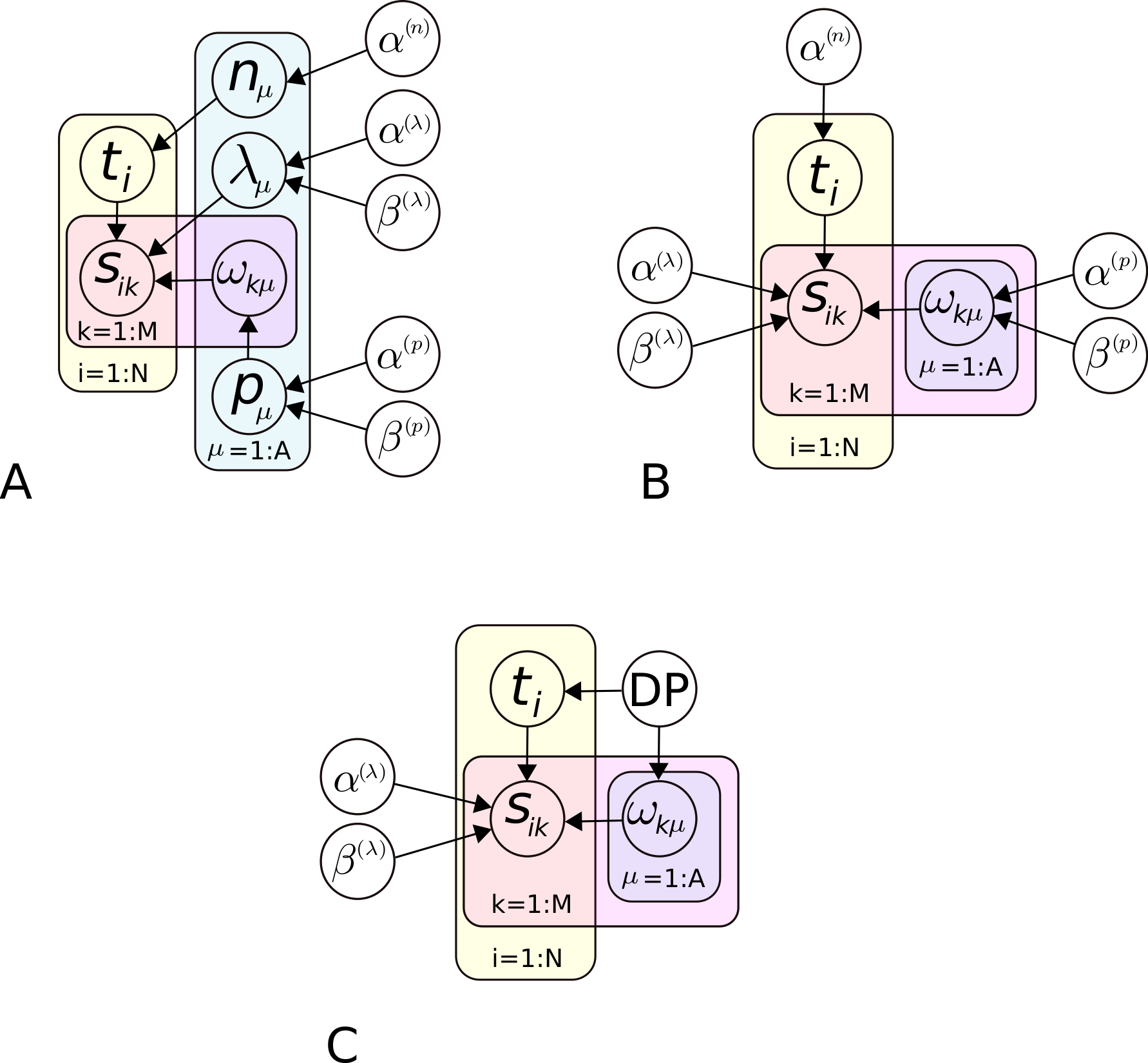

Supplement: S1 Fig — (A) Full model. Nodes in the network represent model variables, and edges denote statistical dependency, (B) collapsed model where assembly activation probability (p) and conditional probability of neuronal activation (λ) are integrated out, (C) collapsed model with Dirichlet process prior on the number of assemblies. (TIFF) [file pcbi.1007481.s001.tiff]

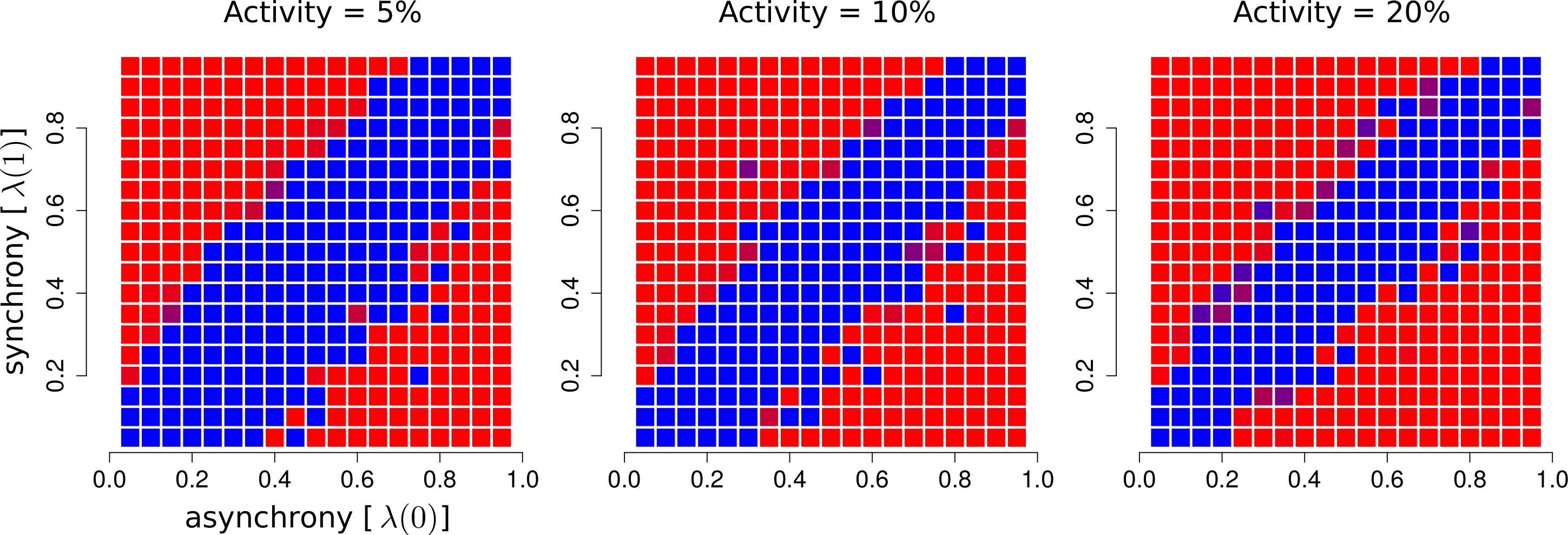

Supplement: S2 Fig — We repeated the analysis represented in Fig 2 to determine the effect of assembly activity on the phase diagram of assembly detectability. When assembly activity increases, the non-detectable regime shrinks towards the line λ(0) = λ(1), corresponding to the limit where the data are non longer informative about neuronal identity. (TIFF) [file pcbi.1007481.s002.tiff]

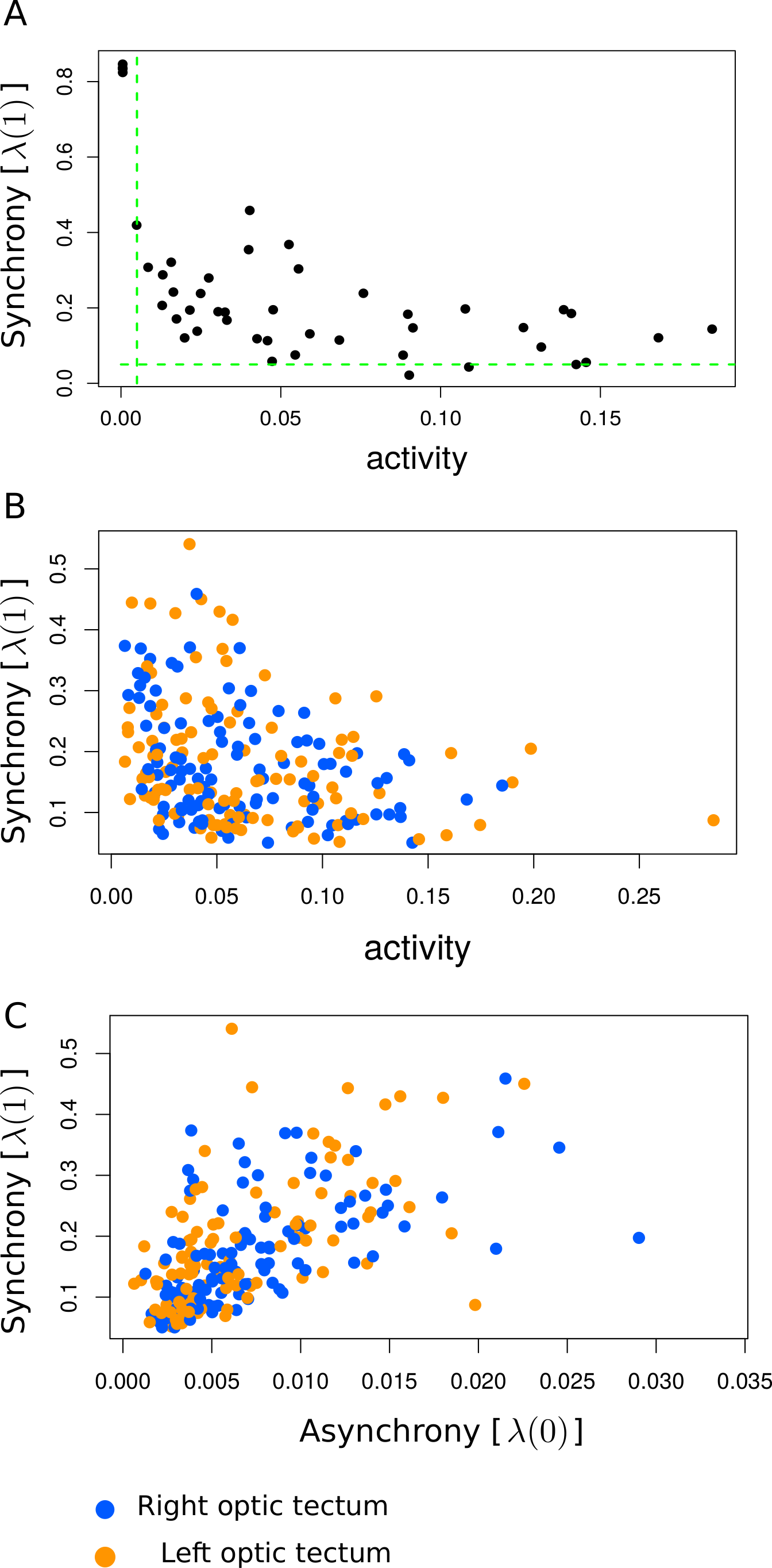

Supplement: S3 Fig — (A) Scatter plot representing the distribution of synchrony and asynchrony levels within one fish. Green (dashed) lines display the thresholds applied to activity (0.005, corresponding to ≈1 event per minute) and coherence (0.05). (B, C) Distribution of synchrony and asynchrony levels versus activity for all assemblies from all fish. (TIFF) [file pcbi.1007481.s003.tiff]

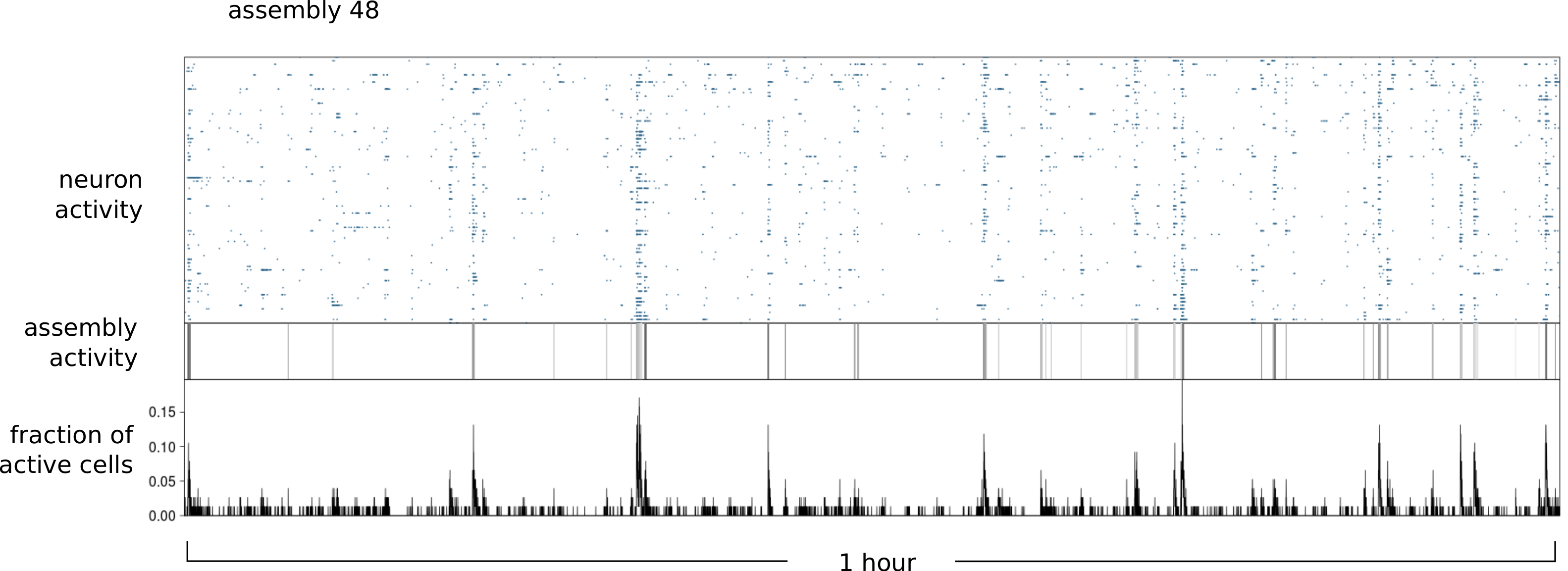

Supplement: S4 Fig — Comparison between the binary activity of all tectal neurons belonging to assembly 48 in Fig 6 (top), the activity of the assembly (middle) and the fraction of active cells within the specific assembly over time (bottom). (TIFF) [file pcbi.1007481.s004.tiff]

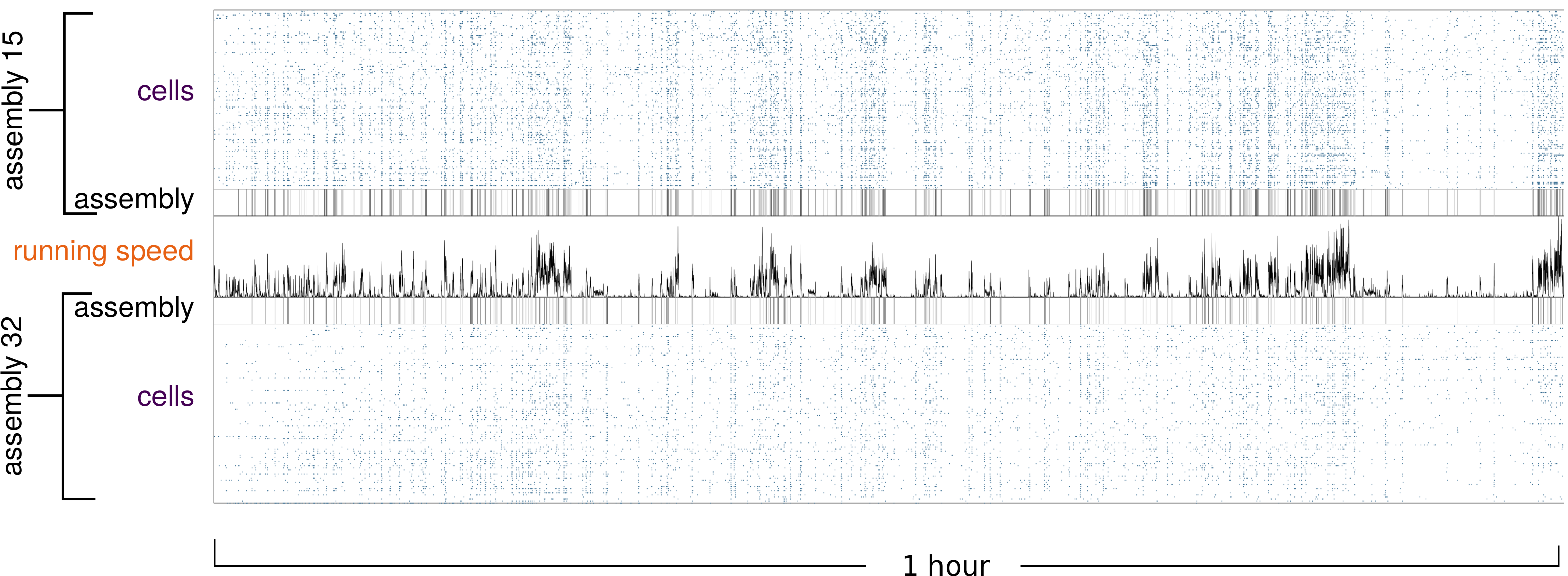

Supplement: S5 Fig — Comparison between assemblies of cortical neurons 15 and 32 from Fig 7. Although both assemblies correlate with running speed, member neurons are separated in two groups due to their different levels of activity, synchrony and asynchrony. (TIFF) [file pcbi.1007481.s005.tiff]

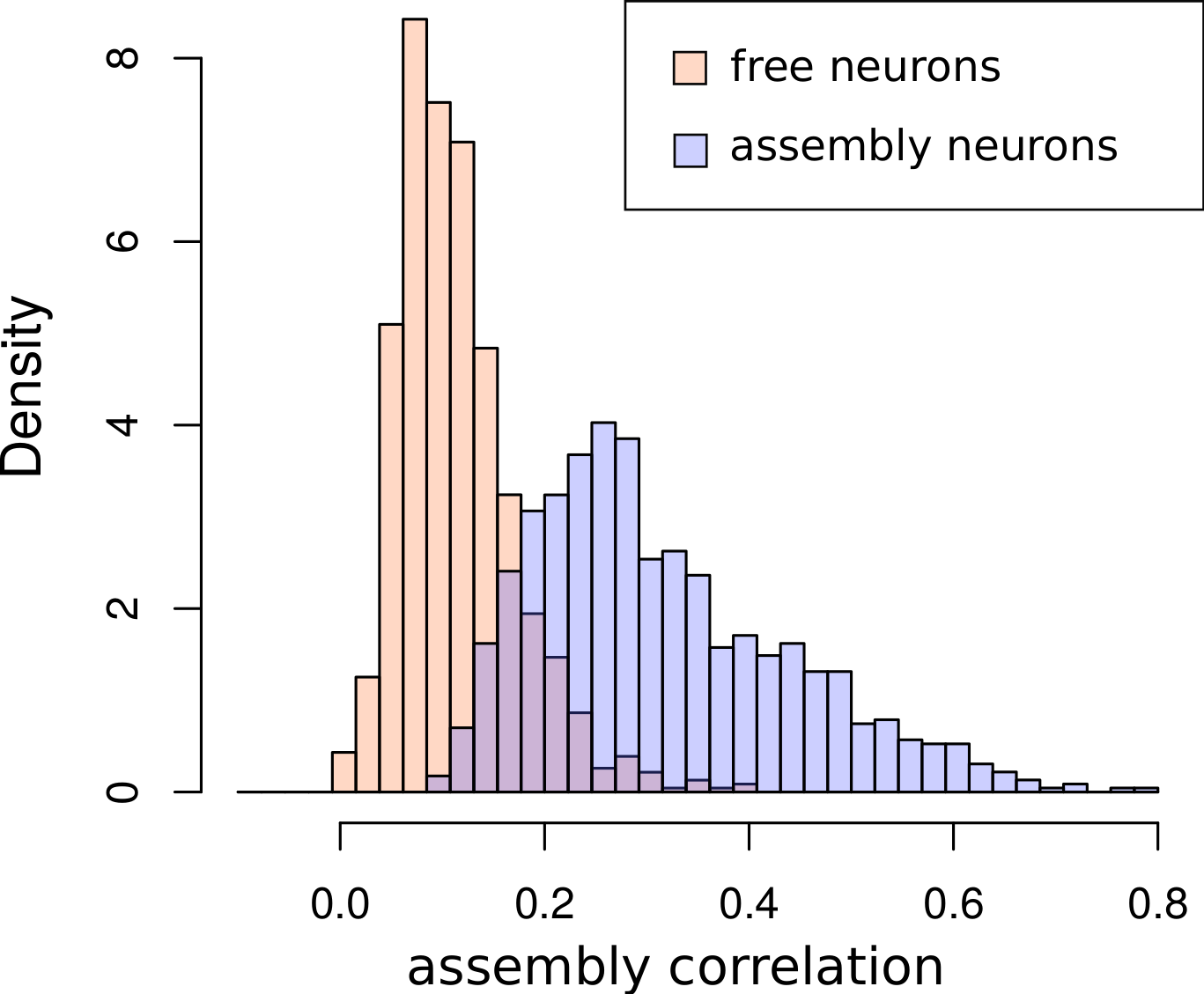

Supplement: S6 Fig — Comparison between the statistics of the time correlation between each assembly neuron and the assembly it belong to (orange), and the maximal correlation between free neurons and the assemblies determined with our method (blue). The correlations of assembly neurons is significantly shifted to higher values with respect to free neuron maximal correlations. (TIFF) [file pcbi.1007481.s006.tiff]

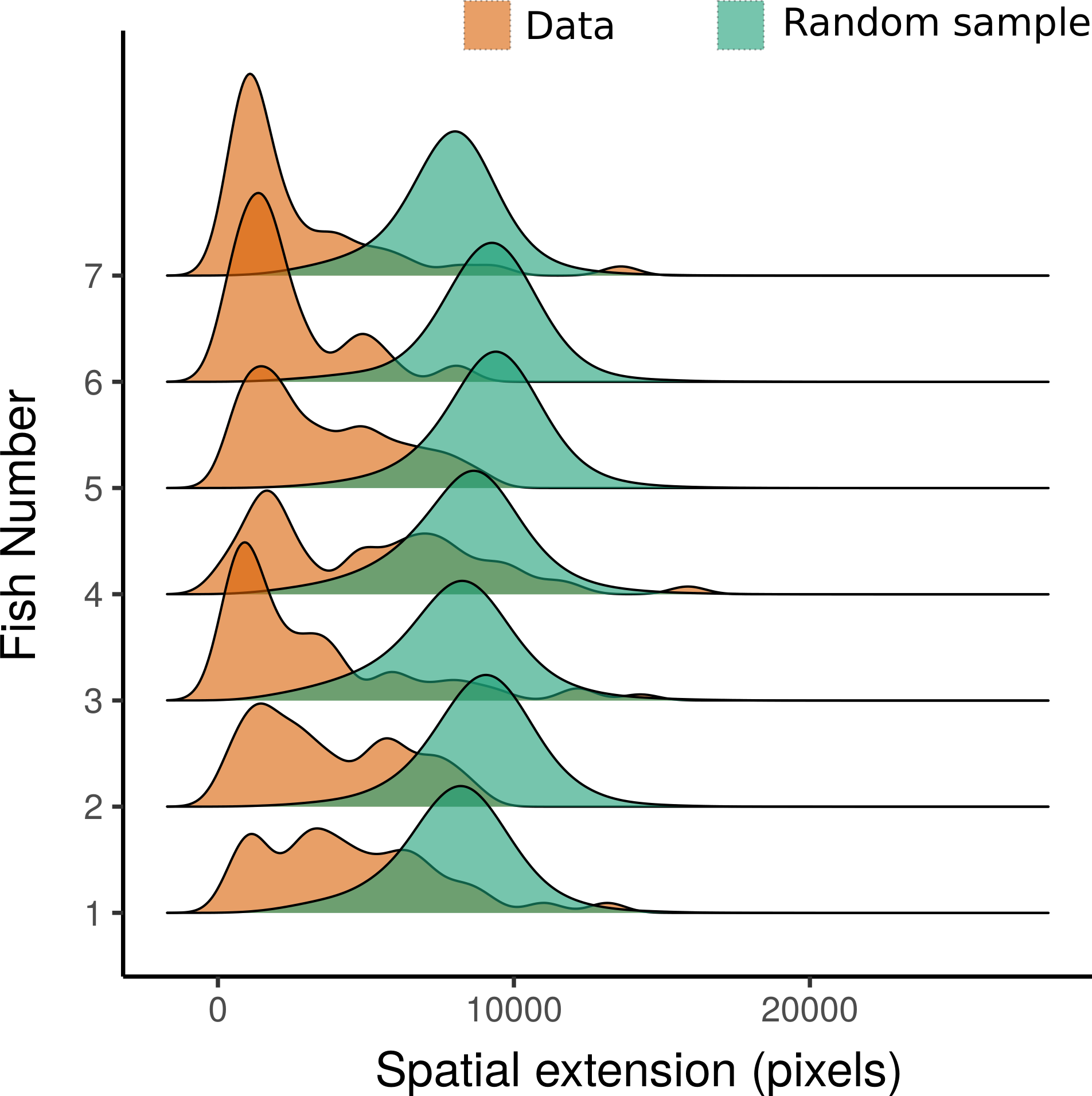

Supplement: S7 Fig — Comparison between the distribution of assembly spatial extension across each fish data (orange) against a null distribution obtained from random groups of neurons of equal size (green). This analysis confirms that tectal assemblies are significantly more compact (reduced extension) than random groups of neurons. The assembly spatial extension was calculated as the area E of the ellipse fitting the assembly 2D distribution, E=πξ1ξ2, where ξ1 and ξ2 are the two eigenvalues of the covariance matrix of the XY neuronal coordinates within the assembly. (TIFF) [file pcbi.1007481.s007.tiff]

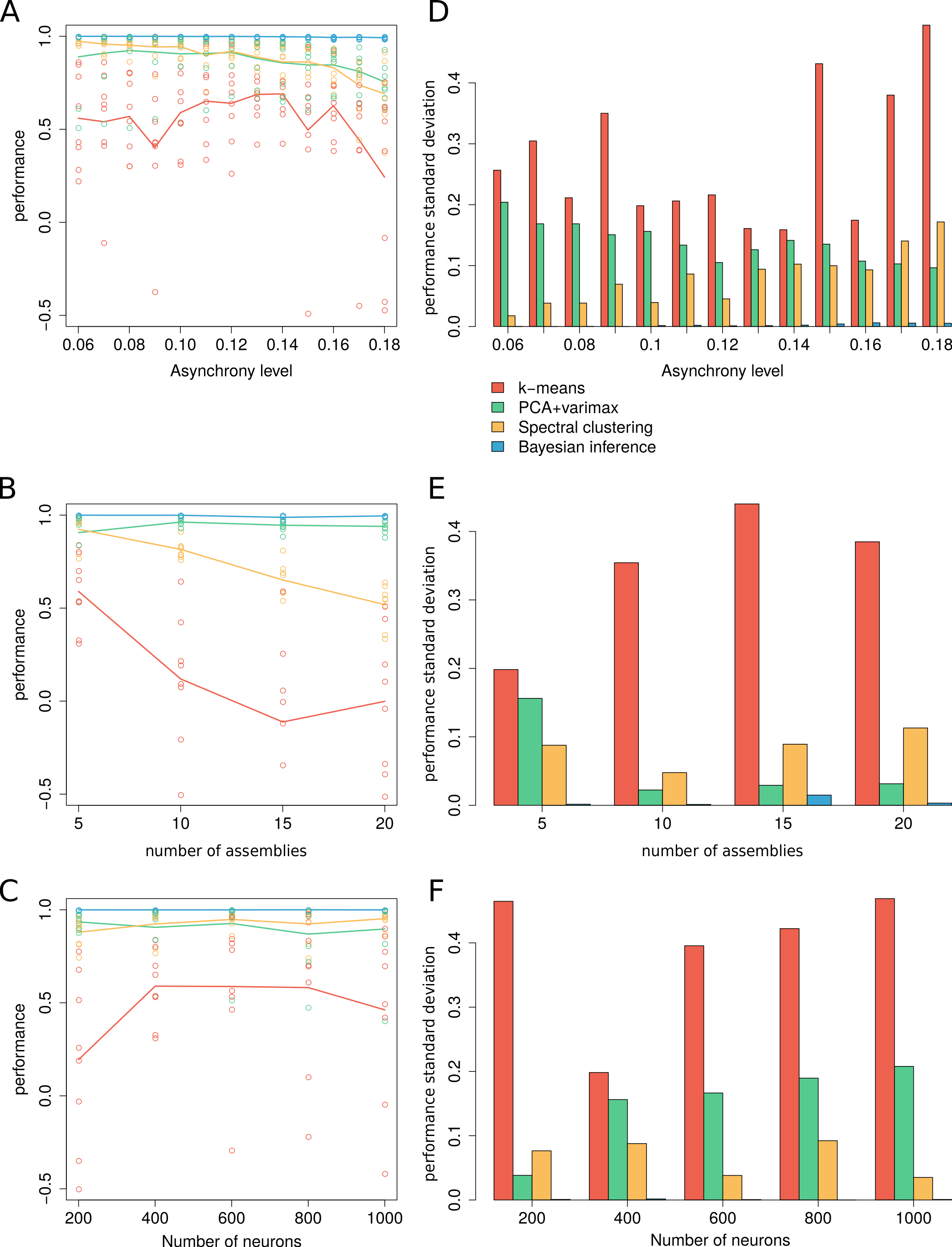

Supplement: S8 Fig — (A) Performance comparison across levels of asynchrony. Dots correspond to independently generated data sets while solid lines show the average performance for each method over all simulated data. (B) Comparison across number of assemblies. (C) Comparison across number of neurons. (D-F) Standard deviation of the performance across simulated data per parametric condition. Unless specified otherwise, surrogate datasets were generated using 400 neurons and 1000 time frames distributed over 5 assemblies with assembly activity of 5%, synchrony 50% and asynchrony 10%. For k-means we used a number of clusters estimated according to the silhouette method. The number of significant principal components in PCA clustering was obtained by the circular shuffling method whereas for the spectral clustering we used the Newman-Reinart graph-theoretic community detection method [22] (see Materials and methods). The performance was measured according to Eq (27) by comparing each set of assignments with ground truth assignments. (TIFF) [file pcbi.1007481.s008.tiff]
