# Supplementary material for: Bayesian inference of neuronal assemblies
Source: PLoS Comput Biol. 2019 Oct 31;15(10):e1007481. doi: 10.1371/journal.pcbi.1007481 (PMC6850560; doi:10.1371/journal.pcbi.1007481)
Supplement: Attachment — Submitted filename: Response.pdf [file pcbi.1007481.s015.pdf]

Reviewer 1

In the manuscript, Diana et al. propose an alternative method for the analysis of neuronal populations based on a Bayesian approach. This technique enables a statistical analysis of neuronal ensemble properties without using dimensionality reduction approaches. The authors used artificial data to validate the methodology and compare with other approaches (k-means, PCA / factor analysis, spectral clustering), and real data obtained from the zebrafish optic tectum and mouse cortex using two-photon calcium imaging and from various regions of the mouse brain using neuropixel electrodes.

This is indeed an additional alternative method among those already developed to study population dynamics in the brain. I am convinced that this approach will be useful for neurobiology labs studying population dynamics. This approach is suitable for a variety of recording techniques and animal models. Although I believe the manuscript is suitable for publication in Plos CB, I have some concerns that need to be addressed:

We thank the Reviewer for their positive comments. We have addressed the concerns raised by the Reviewer and have provided a point-by-point response below. In particular, we have clarified the issues arising from the comparison of different clustering methods. Furthermore, we have tested our method using simulated data generated from an extended model where neurons can belong to multiple assemblies, as suggested by Reviewer 1. Although we did not develop an inference algorithm for this model (see our response for details), we now show that our current method performs well even in the presence of neurons recruited by multiple assemblies.

Comments

1. *In my opinion, comparison of one method with others is always problematic since a method could perform better for a given aspect and be worse for a different one. Also, the authors use different arbitrary parameters and thresholds for each of the different methods. Since the performance of these methods depends on the chosen parameters, the comparison between methods is not very reliable. This counts also for reference [16] which is cited by the authors. So, I would be cautious on how to interpret these comparisons. I believe that there is no need for justification. The decision needs to remain on the potential users who will decide which method will be best for analysing their own data.*

We agree with the Reviewer. It is up to the user to decide which clustering method is better suited for the type of data. In the revised manuscript we modified the text to acknowledge this. However, we believe that some performance testing is required and it has also been requested by other reviewers. Moreover, the hierarchical model introduced in our work uses the most minimal assumptions required to produce assembly-like activity i.e. neurons are assigned to assemblies and they fire in synchrony within their assembly with a certain level of noise. The fact that current methods do not perform well on data generated from this simple model shows their limitations in recovering the basic structure that the user would like recover.

The authors do not mention CPU time of their approach. How long does it take to analyse their data ? It can be done with a regular computer or a computer cluster is necessary ?

The CPU time required to analyze a dataset of approximately 1000 neurons for 1000 time frames is 2-3 hours on a regular desktop machine. All our analyses were done using an iMac with CPU intel(R) core(TM) i7-3770 3.40GHz. Larger data sets with twice as many neurons and time frames can take up to 12h. We have added this to the Methods section (lines 721-725).

2. *line 102. The claim that each neuron belongs to only one ensemble is too strong and not realistic. The authors are aware of this as they explicitly mention it in line 466. I would definitely incorporate their suggestion of introducing latent variables. This could indeed explain why 50% of the neurons in the tectum of the fish behave as isolated neurons.*

The Reviewer raises an interesting point. However, the definition of neuronal ensembles or assemblies adopted in the current literature ('a group of neurons that tend to fire in synchrony') is quite vague. At present, there is no statistically rigorous way of describing how individual neurons participate in population activity. Thus, the statement that each neuron belongs to only one assembly might well be overly simplistic but the fact is that currently we simply don't know. The aim of our method is to address this problem precisely, and we did so by developing a simple model of population activity in which neurons belong to only one assembly. Because we had no a prior information on how the data are structured there was no principled reason to develop more sophisticated models (that include multiple membership, for instance) without first seeing how well the data fit the simple model. Our method allows us to show, with a level of statistical confidence, that there are indeed populations of neurons that only belong to one assembly. Our method also reveals that there are neurons that

cannot be assigned to a single assembly with the same level of confidence. We state the reasons for this in the Discussion of the initial manuscript, one of which is multiple membership.

Although the generative model is easy to generalize to the case of multiple membership (indeed we use it in the revised manuscript to generate testing datasets), extending our current Gibbs sampler to perform inference from this generalized model is non trivial for two reasons: (1) algorithm 2 in the manuscript employs the Dirichlet process to estimate the number of assemblies which strictly assumes single membership; (2) the efficiency sampler is guaranteed by using the "collapsed Gibbs sampler" where the continuous parameters of the model ($\lambda(0)$, $\lambda(1)$ and p for all assemblies) are integrated out. This operation is possible due to the factorization structure of the likelihood which does not hold when neurons are allowed to be recruited by more than one assembly. This is due to terms in the likelihood representing the probability of neurons to be recruited by any of the assemblies it belongs to. For any neuron i these terms have the mathematical form

$$1 - \prod_{\mu : i \in \mu} (1 - \lambda_\mu(\omega_\mu)) \tag{1}$$

which couples synchronous and asynchronous firing probabilities of all the assemblies (μ) neuron i belongs to. For single membership, the terms in Eq. (1) reduce to $\lambda_\mu(\omega_\mu)$, providing a factorized form of the likelihood. This issue makes it impossible to integrate out the λ parameters, which is an essential step to reduce estimation uncertainty and speed up the convergence of the Markov chain.

On the conceptual side, a model where neurons are allowed to belong to more than one assembly introduces ambiguities. In fact, if a group of neurons fires at times where two assemblies are active, the neurons in this group could have dual membership or be considered as a third assembly, which is what our current approach would do. Allowing multiple membership also requires the user to make choices on the logic used by neurons to integrate the activation state of the assembly they belong to. For instance, a neuron belonging to two assemblies might become active when *any* of the two assemblies are active or only when *both* assemblies are active, analogously to an OR and AND gate respectively. These choices would reduce the generality of the method. In the case where neurons with multiple membership were present, our current model would either allocate additional assemblies to accommodate their temporal patterns (which can be studied in a second step of analysis, as we did in Fig. 6C) or assign them to the closest existing assembly with low probability.

So far, none of the methods used to detect neuronal assemblies allows for a statistical distinction between neurons that belong to one assembly and those who potentially might be shared or independent. Our method provides this classification based on probability theory, and generates model-based dimensionality reduction of high dimensional data which guides exploration of the data and generation of new hypotheses. For these reasons we prefer to keep our method as simple as possible. The new analysis presented in our revised manuscript when comparing across different methods shows that our current technique can also deal with scenarios where up to 20% of the neurons have multiple membership, allowing to assign them to one of the assemblies they belong to with an accuracy that for a wide range of model parameters is still superior to other methods even if the assumption of single membership is not satisfied by the test data sets. We have added a section in the discussion of the revised manuscript specifying the workflow of model-based inference needed to extend our current analysis to test different hypotheses.

Do these neurons show pairwise correlations with others? This should be tested to check whether these neurons are indeed isolated form the rest or are excluded because they belong to more than one ensemble.

We performed the correlation analysis suggested by the Reviewer. For one of the data sets analyzed in the manuscript, we have calculated the statistics of (1) the time correlation between each clustered neuron and the assembly it belongs to, and (2) the maximal correlation between non-assigned ('free') neurons and the assemblies determined with our method. In Fig. R1, the correlations of clustered neurons is significantly shifted to higher values (blue) with respect to free neuron maximal correlations. This analysis shows that our classification of

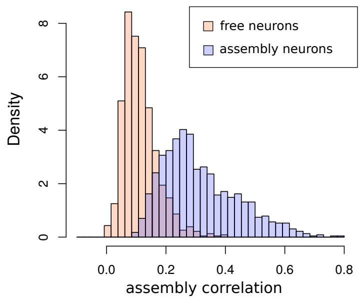

Figure R1: Correlation analysis of assembly and free neurons.

clustered and free neurons is consistent with the data, as free neurons obtained using our method display a

reduced correlation with the existing assemblies compared to the correlation between clustered neurons and their assembly. Figure R1 is now included as a supplementary figure (Fig. S6, see lines 356-357) in the revised manuscript

I would also use synthetic data sets where ensembles will not be discrete (neurons should belong to more than one ensemble).

This is a good point. To address this we employed a different generative model to simulate testing datasets to evaluate the performance of each clustering method (see lines 263-271). As suggested by both Reviewer 1 and 2 we allowed neurons to belong to more than one assembly. In the new analysis presented in Fig. 3 of the revised manuscript we allowed 20% of the neurons to belong to up to 3 assemblies. This leads to a reduction in the performance of all methods. However, even with these new testing data our method outperforms the other methods tested.

3. *All figure legends are very brief, especially those of the supplementary figures. Legends should describe the figures in detail for non-expert readers. Some axes legends are missing. Legend of Figure S6 is missing.*

We have improved the level of description in all figures and fixed missing labels. Figure S6 was a duplicate of Figure S5 and it has been now replaced in the revised manuscript.

4. *Adding an explanatory figure on how the HHM approach for detecting the calcium transients will help better understanding the method.*

We have added a new panel in the revised Fig. 4D to describe the HMM graphical model used.

5. *Fig 5E. is this a stack or a single plane? please specify. Fig. 6F is confusing. Adding just a few it will be enough. The rest can be shown in a supplementary figure.*

Assembly maps are obtained by projecting in 2D the positions of all member neurons across the five imaging planes. We clarified this procedure in the revised manuscript. We thank the Reviewer for the suggestion about Fig. 6F but in our opinion it is important to have all the assemblies displayed in 6F to link the ensemble temporal dynamics (6A), subnetwork analysis (6C) and spatial organization (6F). All panels have the same color code to guide the reader.

6. *Why only time frames with more than 15 active cells were considered? please justify or explain.*

Applying a threshold on the number of active cells is used to 1) reduce the dimensionality of the data to analyze and 2) to focus specifically on events where there are co-active neurons (a signature of ensemble activity). The standard way to obtain such threshold is by shuffling independently each neuron over time and use the statistics of randomly active cells as a null model. From the shuffled binarized activity matrix, we found that the expected number of active cells is ≈ 10, with 2% chance to be larger than 15 consistently across data sets. Applying this threshold reduces the number of time frames to analyze by a factor of 10, reducing dramatically the running time of the inference algorithm. We clarified the origin of the threshold in the revised manuscript (lines 344-346).

7. *explain the rationale of using the parameters in lines 323-328.*

Because all assemblies are defined by the member neurons, in order to accommodate noisy neurons or "free neurons", the Gibbs sampler can generate assemblies with very low activity or levels of synchrony and asynchrony too similar, with the risk of falling in the non-detectable regime. To avoid this we applied the thresholds on the ensemble parameters. The threshold on the level of activity was set to 1 event per minute, according to what has been observed by other authors. The levels of synchrony (asynchrony) were required to be larger (lower) than 5% based on the their distributions across data sets and trials however it remains up to the user to select the thresholds that are biologically relevant.

8. *line 450. The authors claim that the ensembles are compact but they do not quantify compactness.*

In our revised manuscript we included a quantification of assembly spatial extension based on the area of the ellipse fitting the 2D distribution of the assembly. To do so we calculated the two eigenvalues (ξ_1 and ξ_2) of the covariance matrix of the XY neuronal coordinates within each assembly and then quantified assembly extension as the product $E = \pi\sqrt{\xi_1\xi_2}$. The new supplementary figure S7 illustrates a comparison between the compactness distribution across each data set against a null distribution obtained by random groups of neurons of equal size, confirming that assemblies are significantly more compact (reduced extension) than random groups of neurons.

9. *Since this is a methods manuscript, it is important that the reviewers could test the approach. The codes are no available.*

We were planing to make all the software available upon acceptance since the code was not required at the submission, however to allow the Reviewer to test our code we have uploaded our C++ software on the GitHub repository: https://github.com/giovannidiana/BINE. We also added a short documentation on how to install and run the program on testing data.

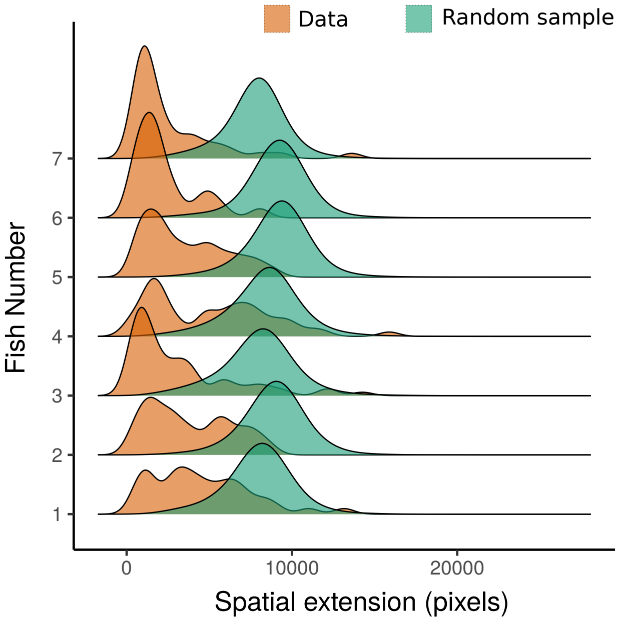

Figure R2: Distribution of assembly extension versus random samples.

Also, it is necessary to clearly and in details describe all the equations and the mathematical procedures. As it is sometimes difficult to follow. This can be done in the supplementary information. For example, in line 203 why $G_\mu^{(-i)}$ *?*

We thank the Reviewer for the comment. To clarify the mathematical presentation we added two sections in the revised Appendix 1 (lines 651-674) where we derive in more detail the calculations for the marginal likelihood integrated over continuous model parameters and the Metropolis-Hastings rule to introduce the Dirichlet process prior. Specifically, the proposal introduced in Eq. 25 (which contains the term referred by the Reviewer $G_\mu^{(-i)}$) was a remarkable result obtained by Neal for which we only provide the reference as a formal proof is linked to the theory underlying the Dirichlet process. We clarified the connection between Neal's result and our application in the revised Appendix and refer to his work for further mathematical derivations (lines 204-210).

10. *Statistics in Fig 3 are missing.*

We corrected a typo in the y-axis label of Fig. 3D-F which is in fact shows the standard deviation of the corresponding performance in Fig. 3A-C for each method calculated across 16 independent testing data sets.

11. *Figure 7A is very confusing. I suggest coloring only neurons that belong to the ensembles shown in Fig 7D and E and Fig S5 and S6.*

This is a good idea. We have revised Figure 7A according to the Reviewer's suggestion

12. *line 336. the temporal resolution was changed from the original recording as only time frames with more than 15 active cells were used for analysis. What's the number of frames excluded?*

Approximately 80% of the frames do not contain relevant assembly events. However due to sustained activity of the population, windows of synchronous activity captured by our method and characterized by subsequent active states in the assembly activity matrix (ω) have the same temporal resolution as in the original recording.

13. *Ensemble usually refers to a group of neurons that could be co-activated by a common source or stimulus. Assembly refers to a group of neurons that are activated together because they are interconnected. Since the authors used spontaneous activity to identify the groups of neurons I would change ensemble to assembly.*

We agree. We have changed the naming in the revised manuscript.

14. *theory. page 3. the notation is confusing.*

Reviewer 2 made some specific suggestions on how to clarify the mathematical notation and presentation of the results. We have implemented these so we hope the manuscript is now clearer.

Reviewer 2

In their manuscript, Diana et al. present a new approach to identify ensemble activity in neuronal population data. Unlike previous methods this approach is purely model-driven and relies on statistical inference using a generative model of ensemble activity. The authors validate their approach on test data generated from their

generative model and test its performance against other methods, claiming that their approach is superior to the others. Finally, they demonstrate the applicability of their approach to calcium imaging data from the optic tectum of larval zebrafish and the visual cortex of mice and show that ensemble activity tends to be spatially localised and that the activity of different ensembles might have behavioural correlates.

Overall, I think the manuscript presents an interesting and promising new method to identify neuronal ensembles in neuronal population activity. I am only concerned about the comparison between the authors' approach and other clustering algorithms that have been applied in a way that seems not always appropriate and fair. Apart from that my remaining comments in the following are mostly about a consistent presentation of the results in terms of terminology and mathematical notation.

We thank the Reviewer for appreciating the novelty of our method and for their comments and suggestions. Following the Reviewer's advice we have significantly improved the manuscript, in particular providing a more appropriate comparison between our inference method and other clustering algorithms. In our revised manuscript we test the performance of all methods using simulated data generated from a different model from the one used for inference. The new testing datasets also include neurons with multiple membership.

Major concerns/comments

1. *Line 256ff. / Figure 3: The results seems to suggest that the proposed approach performs perfect under all conditions. However, to some extend this does not seem too surprising given that the data for the assessment was generated from the same generative model that is then used to fit the data.*

We agree with the Reviewer on the fact that using simulated data generated by the same model used for Bayesian inference will lead to optimal performance. However, we do not imply that our method works perfectly under all conditions. Indeed, we show in the paper the difficulty of decoding the underlying structure of the data near the non-detectable phase (see validation section).

While for a validation this is fine, it seems not fair for a comparison between different methods. The authors would need to show such performance on test data from an independent generative model of ensemble activity.

We agree with the Reviewer. To address this issue we now have changed the generative model used in the manuscript to simulate data in our performance comparisons (lines 263-271). The new generative model is an extension of the one used for inference and it allow neurons to be recruited by more than one assembly. This feature breaks severely the assumptions of our model so in order to have a fair comparison between the four methods used we allowed 20% of the neurons to have multiple membership. With this addition, the performance of all clustering methods decreases systematically. In the range of low asynchrony and number of assemblies, the spectral clustering seems to cope better with multi-membership since the clustering is done on activity frames rather than neurons. In other regimes of the generative model used, our method has higher performance, especially for higher number of assemblies and asynchrony. This is due to the under-estimate of the number of assemblies from the communities of the knn graph representation of the binary data (see our response to point 4).

Also, how where the neurons in the test data distributed into the different ensembles? It seems that the population was simply divided into five (or more) roughly equally sized ensembles. If so, this also raises the question how the performance is affected by having some neurons assigned to individual ensembles and in that sense having neurons that do not contribute to the ensemble activity, i.e. "free neurons" (Figure 5).

This is a good point. In the extended generative model accounting for multiple membership we split the neurons into five (or more) groups with relative sizes randomly drawn from a Dirichlet distribution with concentration parameters set to 2. 20% of the neurons in each group are randomly assigned to a second assembly. Among the neurons belonging to two assemblies 20% of them are also allowed to have a third membership. Neurons can be recruited by any of the assemblies they belong to with synchronous and asynchronous firing probability determined by the recruiting assembly. As suggested by the Reviewer, we tested the effect of having "free neurons" in the simulated data. This was done by setting synchronous and asynchronous firing probabilities to be equal in one of the assemblies (20% of the neurons). This condition allows neurons to fire independently on the assembly state. Introducing such a condition did not have a significant effect on the overall performance (Fig. R2).

2. *Line 584ff.: When k-means clustering was used, was it performed on all activity frames or only on those that had an ensemble event? In case of the former, the frames that do not have an ensemble event could potentially impact the clustering because all the frames have to be assigned to one of the five (or more) clusters. However, in order to produce an optimal clustering of the ensembles there might be more than the assigned number of clusters necessary in order to be able to cluster the frames that contain no information about ensembles away from those that represent ensemble events. Furthermore, the k-means algorithm performs the clustering according to some notion of distance. Which distance was used here? And, given the number clusters that the k-means clustering returned, how was the neuronal identify of the ensembles reconstructed?*

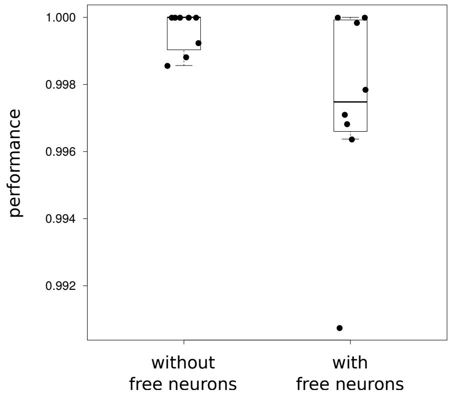

Figure R3: Performance comparison between testing data sets with or without free neurons.

We included all time points when using the k-means algorithm for comparison with our method. However we did not perform k-means clustering on activity frames (as done for the spectral clustering), instead we applied k-means on neuronal activity over time using the standard implementation in R which uses Euclidean distance

$$d^2(n_1, n_2) = \sum_k (n_1(t_k) - n_2(t_k))^2, \tag{2}$$

and neuronal membership assigned to the nearest cluster center. We agree with Reviewer 2 that selecting only statistically active frames could help k-means by reducing the data dimensionality, however since we do not cluster on activity frames, there is no particular reason to expect more clusters than assemblies. Dimensionality reduction is a standard practice when using k-means, however the reason for using original k-means as reference in our comparisons was precisely to illustrate how much the curse of dimensionality affects the performance of standard k-means with neuronal activity data. Importantly, when using our method on simulated data we did not make any selection of time frames, same as used for k-means.

3. *Line 590ff.: When applying the PCA method to infer ensembles, a varimax rotation was used as opposed to the promax rotation (cf. Romano et al. 2015). Why did the authors choose to do so?*

Varimax rotation has also been used by other groups (cfr. Hamm et al, Neuron 2017) for detecting neuronal ensembles. Although for real data factor correlations could require the use of non-orthogonal rotations, neuronal activity in our simulated data is sparse and assemblies are rarely coactive, therefore we expect explanatory factors not to overlap significantly, which justify an orthogonal rotation (varimax) on the principal components. In general, non orthogonal rotations could improve the performance of PCA-based methods when correlations are significant, however it is the number of ensembles that more significantly impacts their performance.

Also, when assigning the ensemble membership there might be neurons that have a very low loading in every of the varimax-rotated components. Rather than being forced into an ensemble defined by a component, they should be considered independent.

We agree with Reviewer 2. We repeated this analysis by considering only neurons with the largest loading one standard deviation above the average. We revised the manuscript accordingly (line 605).

4. *Line 601ff.: When applying the spectral clustering method to infer ensembles, it seems that the last step in the procedure is missing in which given the clusters from the spectral clustering activity core ensembles were constructed and frames were reassigned to different clusters (cf. Avitan et al. 2017). Why did the authors choose to do so?*

In the original pipeline proposed by Avitan et al. 2017, after an initial estimation of the core assembly activity patterns, the authors introduce a refinement procedure where patterns are filtered based on size and then merged up to some similarity threshold to allow good separation between assemblies. This step is then followed by the reassignment of all synchronous activity frames to the new core assemblies. When applying Avitan's method to real data the refinement procedure is important due to the high level of similarity between sequential activity patterns obtained from calcium imaging data, which gives rise to an overestimate of the actual number of assemblies. However, for the data simulated using our generative models where activity patterns are not temporally correlated, we have found the opposite effect where the number of assemblies is often underestimated by the communities in the k-nearest-neighbor graph. For this reason, the merging steps would not be appropriate for our simulated data since it would reduce the overall performance of the method.

Similarly as in PCA clustering, there might be neurons that have a very low affinity to some of the clusters. However, the procedure chosen here forces them into an ensemble although they should be really considered independent.

We agree with the Reviewer. We have repeated the analysis and considered as independent all neurons with affinity (as defined in Avitan et al. 2017 and M olter 2018) lower than 20% as now described in the revised manuscript (lines 710-711).

Minor concerns/comments

1. *Line 39f.: It is stated that in PCA-base methods the number of ensembles "is equal to the number of principal components required to explain the variance of the data". However I believe that in these methods the question is not really about how much variance is explained by including a certain number of principal components. Rather in these methods ensembles correspond to principal component that cannot be explained by chance, i.e. if there were no temporal correlations between the neurons beyond those that are present given the overall activity level alone (cf. Lopes-dos-Santos et al. 2013).*

   We agree. We clarified this point in the revised manuscript (lines 40-42).

2. *Line 52: "a-priori" should be spelled as in the remaining part of the manuscript, "a priori" (Line 94).*

   We have corrected this typo in the revised manuscript.

3. *Line 101f.: "We assume that each neuron belongs to one ensemble [...]" - Clearly, in every population there are neurons that do not participate in the ensemble activity in the sense that they largely act independently. I believe that the authors suggest that such neurons are part of their individual ensembles. However, since one could argue that an ensemble requires at least two members, it might be good to mention explicitly that ensembles in their model can also consist of single neurons.*

   We have clarified this point in the new manuscript, also emphasizing that neurons can act independently while still be grouped into an assembly when the difference between synchrony and asynchrony of that assembly is low, in which case the state of the member neurons is independent of assembly state (lines 352-354).

4. *Line 112ff.: The definition of the ensemble synchrony/asynchrony seems to be uniform across the neurons of this ensemble. However, some neurons might be more reliably activity given the ensemble is active than others and in that sense have a higher affinity to the ensemble (cf. Thompson, Scott 2016). Therefore, how would this assumption affect the applicability of the approach?*

   We appreciated the Reviewer's comment on the model. We addressed this in the revised manuscript. As the Reviewer correctly pointed out, assembly synchrony and asynchrony are uniform parameters across the neurons in the assembly as they are meant to characterize assembly activity and not single neurons. These parameters characterize respectively the "average" levels of coherence and noise in the assembly activity. However, with our approach we can estimate the probability of each neuron to belong to each of the assemblies. Neurons which are more synchronous with their assemblies will be characterized by higher probabilities whereas neurons more sporadically recruited by their assemblies will have a lower assignment probability. Having neurons with higher and lower affinities is not a problem for our method since we can use neuron-specific posterior probabilities to discriminate between neurons with higher and lower affinities to the assembly. Moreover, the user of our method can also compare the time correlation between each neuron within an assembly with the assembly activity (given by the ω matrix) to inspect the affinity of each neuron to its assembly.

5. *Line 122f.: The terms "coherence" and "noise" are not defined and seem to correspond to the previously introduced terminology of "synchrony" and "asynchrony". This should be fixed.*

   The reviewer is correct. This has been corrected in the revised manuscript.

6. *Equations (4), (5): It seems that the shape parameters do not depend on the ensemble. However, later when doing the inference (Equations (21)-(23)), there is a differentiation between shape parameters for the different ensembles. Hence, if the authors assume different shape parameters for different ensembles, they should indicate that also in Equations (4), (5) and add the corresponding subscripts.*

   We added the new notation in the revised manuscript.

7. *Equations (11)-(13): To make the relation of these definitions to the definitions above (Equation (9)) clear, the authors should also state the dependence on z and z' explicitly.*

   We have clarified the link between Eqs. 11-13 and Eq. 9 in the new manuscript. As adding other indeces zz' would make the notation heavier we explicitely mentioned in the text that we use matrix notation (lines 150-151).

8. *Line 152f.: Similar to the way it is mentioned before, it should be stated that* $T^{zz'}_{S;\mu}$ *also only counts certain events "up to an additive constant".*

   We clarified this in the revised manuscript (line 155).

9. *Algorithm 1, 2: In order to the denote the ranges the different parameters are chosen from, the same notation as in the rest of the manuscript should be used, i.e.* $\mu \in \{1, \cdots A\}$ *etc.*

   We modified the revised manuscript accordingly.

10. *Line 218f.: It is stated that ensemble labels are initialised uniformly to values "between 0 to* A^{max} *where* $A^{max} \approx N$*". First, I believe the range is given by 1 to* A^{max}*, because by definition 0 is not an admissible label (Line 103). Second, it is not clear what range from which the labels are drawn actually is. I believe that it should maybe say "*$A^{max} = N$*".*

    We clarified this point in the new manuscript (lines 224-225).

11. *Figure 1: "Cells assigned to* $A \approx N$ *ensembles are [...]" - It is very unlikely that the initialisation procedure (i.e. drawing labels independently and uniformly for the range of 1 to N) as described before results in approximately N different ensembles. In fact, in expectation there will be* $N * (1 - (1 - 1/N)^N)$ *ensembles which for N sufficiently large means that* $A \approx 0.63N$*.*

    We introduced the notation $\mathcal{O}(N)$ to clarify this point in the new manuscript (line 225).

12. *Figure 2 and Figure S2: The relation between synchrony and asynchrony for the recovery of the ensembles is discussed in the main text and the caption. However, in the figure the axis labels read "Ensemble coherence" and "Noise". This should be fixed. Also, I might be helpful to include the corresponding symbols* $\lambda(1)$ *and* $\lambda(0)$ *in the labels in parentheses.*

    We modified Figures 2 and S2 according to the Reviewer's suggestion.

13. *Figure 3: The variability in the performance is shown in panels D-F and not only D-E. In addition, while the axis labels read "Performance variance", in the caption this is referred to as the "standard deviation". What is actually shown?vThere are characters missing in the axis labels in panel E and F.*

    We updated Figure 3 in the revised manuscript using data simulated from the new generative model which allows neurons to belong to multiple assemblies. We also corrected the typo in the axis labels and the caption according to the Reviewer's comment.

14. *Figure 4: The caption for panel A distinguishes between a left and right part. However, the system setup is not shown in panel A.*

    We corrected Figure 4 according to the Reviewer's suggestion.

15. *Figure 5: Unlike in the caption, the axis labels in panel D read "coherence" and "noise" which should be "synchrony" and "asynchrony" as before.*

    We corrected typos in Figure 5 according to the Reviewer's suggestion.

16. *Figure 6: In the network in panel C some edges are drawn in red. What is their relevance? When measuring the physical distance between neuronal ensembles, how is this done?*

    Red edges denote connections between nodes across communities. To relate physical distances and correlations among ipsi/contra lateral assemblies we focused on planar distance between assembly centers obtained by averaging the 2D coordinates of member neurons on the imaging plane. Distances in pixels were converted into microns according to the scaling shown in Fig. 4B (bottom panel), corresponding approximately to 0.9μm per pixel. We clarified this point in the revised Figure 6.

17. **Figure 7: What is the temporal scale in panels B and D?**

    The recording was approximately 1h long. We specified the overall duration in the revised figure.

18. *Line 416f.: The HMM method to detect the onset of calcium transients was also used to detect the onset of increases in the firing rate. However, the HMM method explicitly assumes an exponential decay of the calcium concentration (Equation (31)). Since the firing rates probably do not have this characteristic, how is this method still applicable?*

    The Reviewer is correct in saying that the HMM method used to extract the onset of calcium transients uses an exponential decay. When binning spike times from neuropixel data we observed that transients of increased firing rate were characterized by a fast activation and slower decay, which is why we used the same exponential model to describe transients of high firing rate. Although the underlying process is different in nature from the GCaMP dynamics we could use the same HMM model by adapting the decay

constant to neuropixel data, which turned out to work very well in extracting high activity time points. Although we used our HMM for both calcium imaging data and neuropixel recordings, the binarization technique is independent of the applicability of our method for detecting assemblies, which gives the user the freedom to employ techniques better suited to binarize their neuronal recordings. We clarified this point in the revised discussion (lines 519-525).

19. *Line 560ff.: In the interest of clarity in the presentation, I would suggest that the notation matches the one in the main text. In particular, this means that time is indexed by k running from 1 to M.*

    We added the suggested notation in the revised manuscript.

20. *Equation (29): The parameter q seems to be undefined. How is it chosen? Also, from Equation (37) it seems that it should actually be* $s_t \sim \text{Bernoulli}(q\delta t)$.

    q is the rate of the Bernoulli process. Its value was initially set to 0.1 and then updated as the other model parameters through the plug-in method discussed at the end of the revised section. We clarified the role of q, corrected Eq. 29 and added the q update rule in the revised manuscript (Eq. (49)).

21. *Equation (31): The parameters* λ *and* δt *seem to be undefined. How are the chosen? It is only later (Line 571) mentioned that* λ *is the calcium decay constant.*

    λ is the exponential decay of the calcium transient and it was chosen to match the typical time scale of the shortest transient, δt is the time step, i.e. the inverse of the acquisition frames per second. We clarified this in the revised manuscript (lines 616-620).

22. *Equation (32): I believe it should be a lower case* x_t *given this is how the variable is used mostly throughout this section. However, this should also be fixed in the other instances where the variable is used upper case to be consistent. Also, instead of* $=$ *it should be* $\sim$.

    We corrected the typo in Eq. (32) and changed the notation from upper to lower case in the revised manuscript.

23. *Equation (36): Given the definition in Equation (31) and e.g. Equation (42),* δt *should not appear in the variance.*

    We corrected the typo in Eq. (36) in the revised manuscript.

24. *Line following Equation (38): The derivative should be taken from* g_0.

    We corrected the typo in the revised manuscript.

25. *Figure S3: The terms "coherence" and "noise" need to be replaced with "synchrony" and "asynchrony"*

    We corrected the typo in the revised manuscript.

26. *References: The author might want to consider revising the references as some author names are misspelled and some DOIs do not show fully qualified DOIs but rather DOI URLs.*

    We apologize for the typos. We revised all references in the new manuscript.

Reviewer 3

In this work the authors describe a new algorithm to identify the neural ensembles in neurophysiological data. They achieve this by inverting hierarchical generative model of ensemble activity by performing a model-based Bayesian inference sampling procedures. They use this to effectively infer ensemble composition as well as the activity in each ensemble e.g the numbers of ensembles and levels of synchrony. They demonstrate the robustness of this algorithm in a simple simulation model, benchmark it against a prominent existing technique as well as apply it to imaging data in fish and mouse as well neuropix neurophysiological data. The method captures known structure in the data in the data as well as revealing interesting new structure. A Bayesian approach to this problem approach to ensemble identification seems long overdue and this paper makes a welcome addition to the literature. The authors also provide code to implement their algorithm making it of immediate relevance to a large community of researchers thus I would like to see the publication of this paper expedited. I have no substantive criticism but below I outline few point clarification and correction.

Thank you for the positive feedback. We have addressed the Reviewer's comments below.

1. *In the body and the conclusion the authors say "arbitrary definitions of time frames that characterize synchrony are not required because our method considers sustained periods of activity by extending the*

time window in which an ensemble is active." It is not clear whether this is part of the inference algorithm or the HMM model they use to identify the events. Please clarify.

This is part of the inference algorithm. Although the model assumes subsequent activity patterns to be independent and identically distributed, the estimated assembly activity from the posterior distribution displays time correlations accounting for sustained periods of activity. This effect very naturally arises as the optimal way to adjust assembly activity according to neuronal activity, with the effect of extending the activity window of an assembly if necessary to accommodate the activity of member neurons. We specified this point in the revised manuscript (lines 373-378).

2. *Relatedly, it would been nice to demonstrate, or at least comment on, the the robustness of the method to the way the data is preprocessed.*

   This is an important point. The preprocessing of neuronal recordings is a crucial part of every pipeline of analysis. Our preprocessing consists of the following steps: (1) image registration, (2) segmentation, (3) signal binarization using the HMM. Since our inference method uses binary neuronal activity as input to detect assemblies, the user can employ any suitable technique to extract binary time series (deconvolution, non-negative factorization, or other model-based approaches) as long as the onsets of increased activity (indicating the start of reverberating assembly activity) are reliably detected from each neuronal time series. Although having a good binarization algorithm is important, our inference algorithm can cope with preprocessing errors (missing or erroneous calcium transients) by adjusting assembly synchrony and asynchrony levels. The robustness with respect to the binarization step is due to the large number of active neurons during an event of synchronous activity. In fact it is unlikely that the binarization algorithm will miss a transient from the majority of the neurons.

3. *Equation 2: Please define z explicitly here.*

   We have defined z in the revised manuscript

4. *One general point, which I think goes beyond the scope of what is achieved here, is how useful is it to describe neural activity in terms of ensemble dynamics. This idea is pervasive in the neuroscience community but has its roots in the theory and ideas of coupled oscillators. It is interesting the authors find many cells that are not easily be assigned to an ensemble. Could the authors comment on the possibility that this is because the neural dynamics are not well described by ensemble dynamics.*

   This is a very interesting point. In the discussion we mention some potential explanations of the large number of "free" neurons (i.e. not assigned to an assembly). One point that we did not discuss is whether, in light of the free neurons, it simply isn't useful to describe neural activity in terms of assembly dynamics. However, we feel this point would need expanding upon, and we're inclined to agree with the reviewer that it is beyond the scope of this manuscript.
